# A Case of Epithelioid Angiosarcoma Diagnosed From Gross Examination of a Pulmonary Tumor Utilizing Imprint Cytology and Immunocytochemistry

**DOI:** 10.1002/cnr2.70014

**Published:** 2024-10-23

**Authors:** Tatsuya Mori, Keishi Mizuguchi, Chie Shimaguchi, Kaori Sakano, Tsubasa Shimoda, Urara Okawa, Miyu Okuda, Mayo Usui, Hiroko Ikeda

**Affiliations:** ^1^ Department of Diagnostic Pathology Kanazawa University Hospital Kanazawa Japan

**Keywords:** angiosarcoma, cytopathology, epithelioid, immunocytochemistry, touch‐imprint

## Abstract

**Background:**

Angiosarcoma, a very rare malignant tumor constituting 2%–4% of soft tissue sarcomas, manifest in diverse organs including skin, soft tissues, and bones. Histologically, angiosarcoma presents a wide range of morphologies, with epithelioid angiosarcoma (EAS) resemblance to carcinoma. The difficulty arises from the shared epithelial‐like morphology and expression of epithelial markers in immunohistochemistry.

**Case:**

This study reports a case where EAS diagnosis was achieved through a combination of gross findings in a lung resection sample, imprint cytology, and immunocytochemistry. Imprint cytology revealed clusters of epithelioid cells, while immunocytochemistry showed positive results for CD31, ERG, Fli‐1, and AE1/AE3, proving instrumental in diagnosing EAS. The described immunocytochemical protocol facilitates prompt diagnosis exclusively through cytology samples.

**Conclusion:**

This report emphasizes the potential for diagnosing EAS using cytological specimens, which is especially useful in cases where obtaining tissue samples proves challenging.

## 1 Introduction

Angiosarcomas represent a rare category of malignant vascular neoplasms, comprising less than 2%–4% of soft tissue sarcomas, officially recognized as a distinct entity in the 2019 World Health Organization classification of tumors of soft tissue and bone [1]. These tumors can manifest in cutaneous sites, soft tissues, and various organs including bone [2, 3]. The histologic presentation of angiosarcoma is highly variable, featuring diverse growth patterns such as papillary, spindled, and epithelioid morphologies [4]. Particularly, a distinct morphologic subtype of angiosarcoma, referred to as epithelioid angiosarcoma (EAS), is characterized by malignant endothelial cells, primarily exhibiting epithelial characteristic [5]. EAS poses a diagnostic challenge due to its morphologic resemblance to carcinoma [6]. Furthermore, vascular tumors such as angiosarcoma often contain a significant amount of blood, complicating the acquisition of sufficient tumor cells for a definitive diagnosis through small preoperative biopsies [7]. Conclusive diagnosis based on cytological material is similarly challenging [8]. While there are reports in the literature of cytological findings or immunocytochemistry (ICC) of EAS, there are no reports of cytology leading to an earlier diagnosis than traditional pathology. One contributing factor to the delay in rapid cytological diagnosis might be the absence of detailed immunocytochemical staining protocols in the literature. The immunostaining protocol for EAS presented in this study addresses this gap, enabling a diagnosis using solely cytological specimens.

In this report, we present a case of EAS diagnosed through cytomorphological features and ICC, employing touch imprint cytology prompted by the gross findings observed in a lung resection sample.

## 2 Case

**FIGURE 1:**
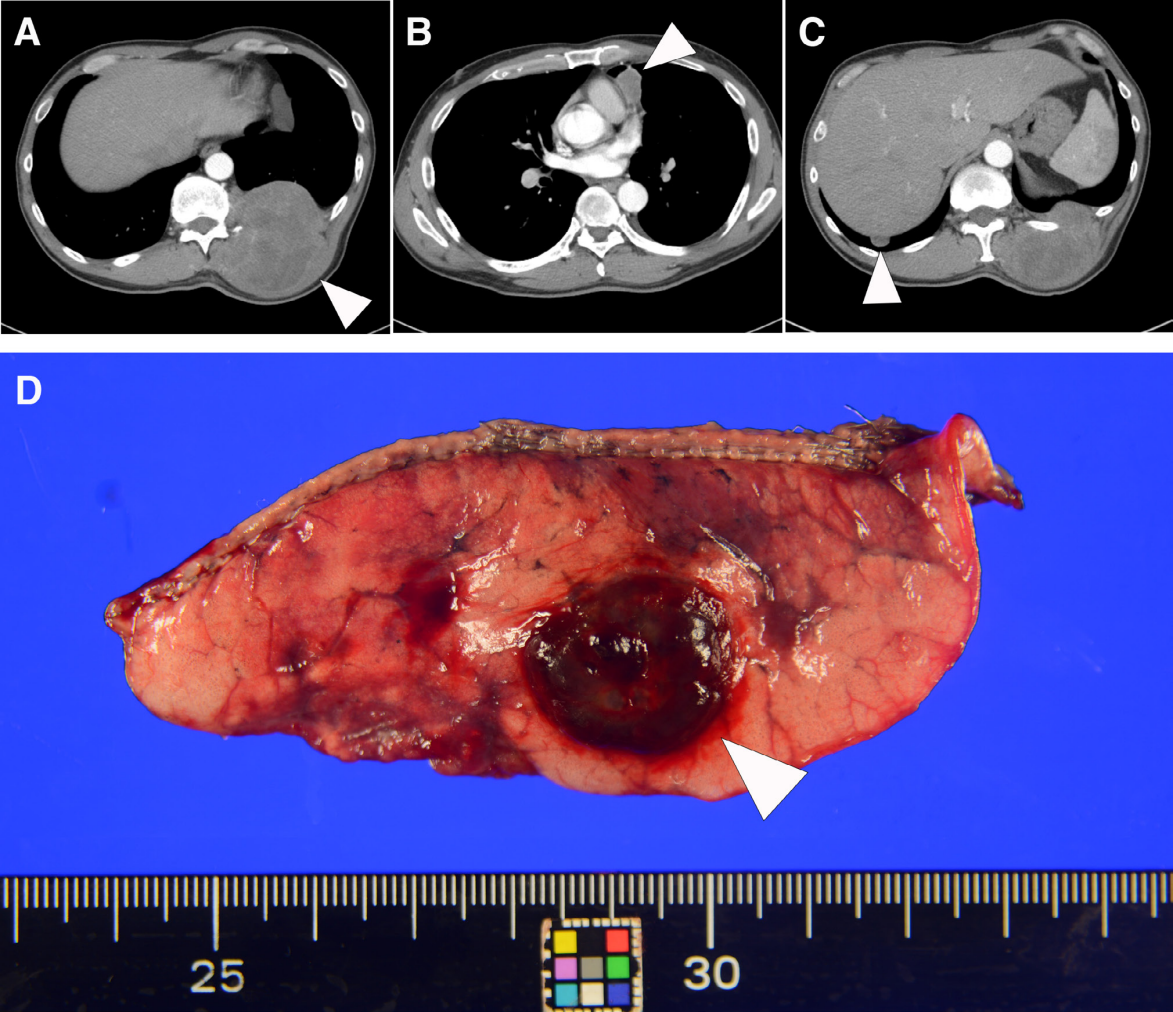
(A–C) Contrast‐enhanced computed tomography. (A) Osteolytic tumor with a diameter of 73 mm on the left 11th rib (arrowhead). (B) Tumor in the mediastinum (arrowhead). (C) Tumor in the lung (arrowhead). (D) Resected lung displaying a dark reddish‐brown hemorrhagic nodule (arrowhead).

A 48‐year‐old male was admitted to Kanazawa University Hospital on January 9, 2020 due to an enlarged and painful mass on his left back. He had been experiencing discomfort without pain or visible abnormalities in his left dorsal region for over 10 years. Recently, he noticed a mass and pain in the left dorsal region. A contrast‐enhanced CT scan revealed osteolytic changes on the dorsal aspect of the left 11th rib, along with a substantial mass measuring 73 mm. Additionally, masses were detected in the mediastinal and lung areas (Figure 1A–C). Considering the clinical course and imaging findings, potential differentials included pulmonary metastases of tumors in the ribs, multiple metastases of lung cancer, and multiple metastases of cancer of unknown origin.

The patient underwent an echo‐guided percutaneous biopsy of the back tumor. Histological examination revealed a few atypical cells with poor connectivity against a necrotic background. Immunostaining results showed positive expression for AE1/AE3 and CK7, with focal positivity for CAM5.2. However, they tested negative for EMA, Desmin, CD34, S100, and TTF‐1. Consequently, based on these findings, the biopsy diagnosis raised suspicion of metastasis from an undifferentiated carcinoma of unknown origin.

To investigate the possibility of primary lung cancer, the patient underwent a partial resection of the lower lobe of the right lung. The gross examination of the resected lung revealed a dark reddish‐brown hemorrhagic nodule (Figure 1D). Considering the gross findings, we contemplated the potential for a vascular neoplasm and proceeded to prepare a stamp‐imprinted cytology specimen of the tumor. The neoplasm was stamped onto glass slides and fixed in either 95% alcohol or air‐dried. The alcohol‐fixed slide was treated with Papanicolaou stain, while the air‐dried slide was treated with Giemsa stain.

**FIGURE 2:**
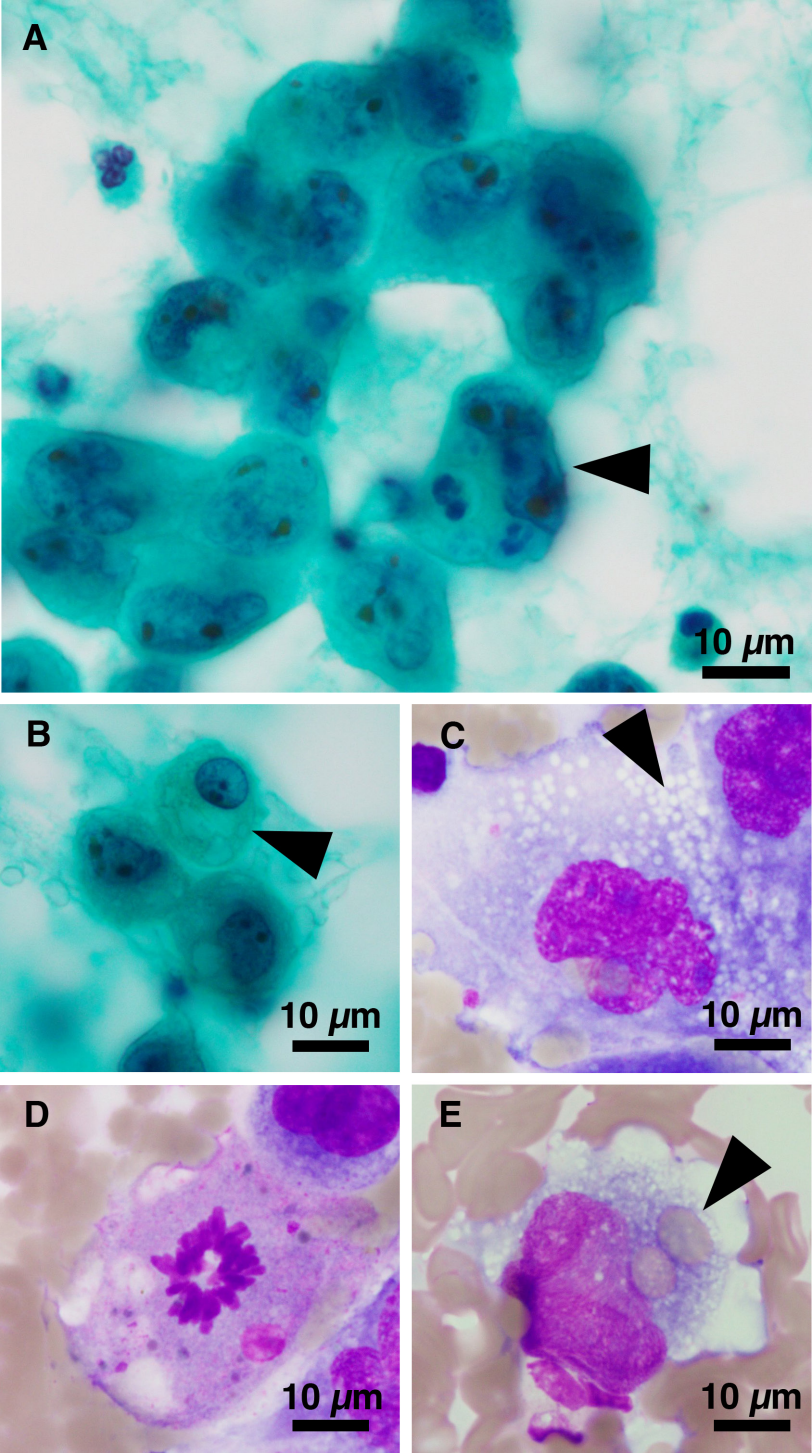
Cytological findings of stamp‐imprinted specimen in lung tumor. (A) Cell clusters with rosette‐like structure. Atypical cells with pleomorphic nuclei and prominent nucleoli (arrowhead). (B, C) Atypical cells with fine to coarse vacuolated cytoplasm (arrowhead). (D) Mitosis. (E) Erythrophagocytosis (arrowhead). (A–C: Papanicolaou. D, E: Giemsa).

Cytological analysis revealed the presence of small to medium‐sized clusters and isolated scattered atypical cells on a bloody background. Some of these clusters exhibited rosette‐like structures (Figure 2A). The atypical cells exhibited an epithelioid or plasmacytoid morphology with fine to coarse vacuolated cytoplasm (Figure 2B,C). The nuclei demonstrated pleomorphism, characterized by gyrate nuclei, and prominent nucleoli (Figure 2A–C). Additionally, some atypical cells exhibited mitosis (Figure 2D), and the cytoplasm contained erythrocytes, a phenomenon known as erythrophagocytosis (Figure 2E). The cytological findings collectively suggested the type of tumor was an epithelioid vascular neoplasm. Moreover, the highly atypical morphology and observed mitotic activity raised suspicion of EAS, with adenocarcinoma considered as a potential differential diagnosis.

**FIGURE 3:**
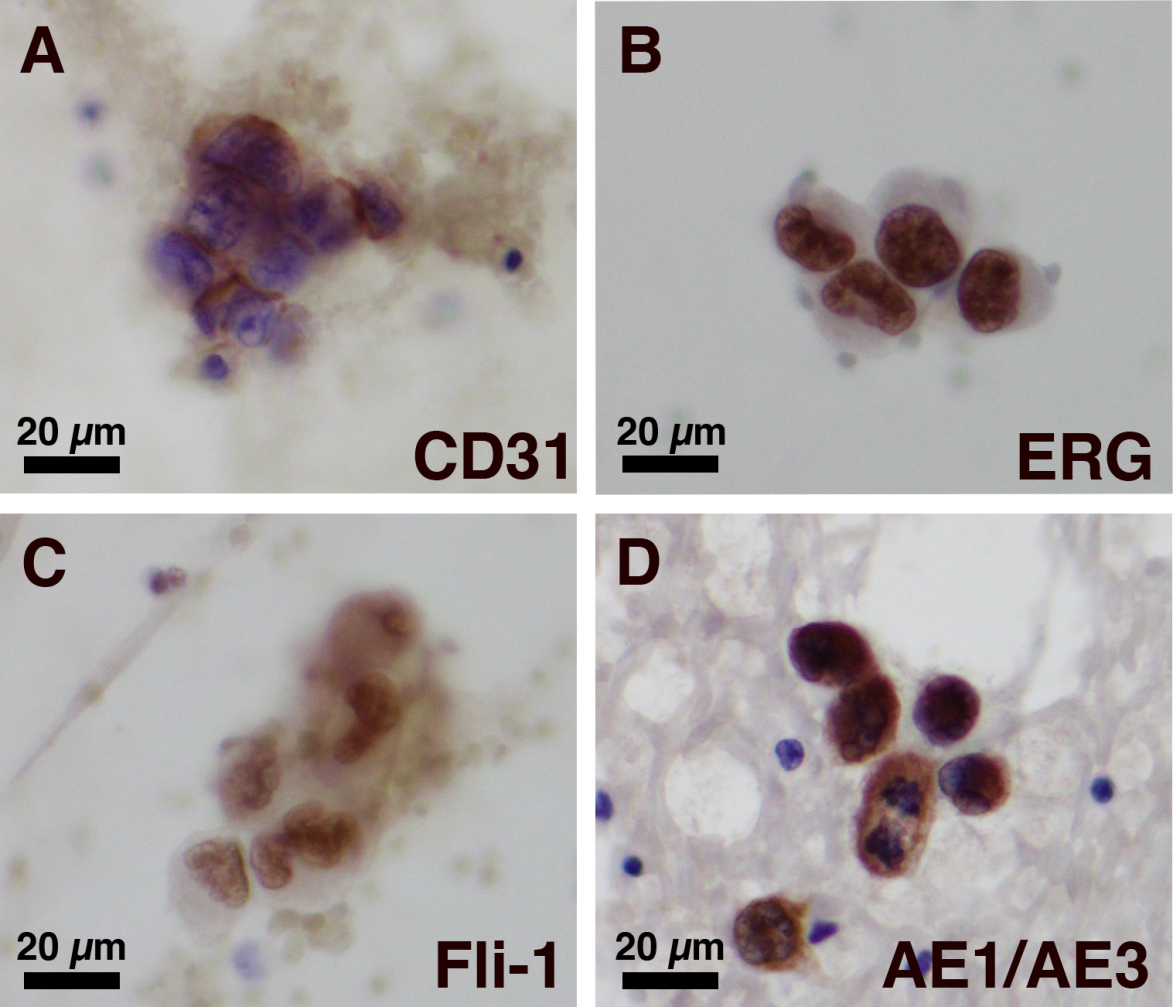
Immunocytochemical findings. CD31 stain showing cell membrane positivity (A). ERG stain showing nuclear positivity (B). FLI‐1 stain showing nuclear positivity (C). Cytokeratin AE1/AE3 stain showing cytoplasm positivity (D).

To distinguish angiosarcoma from adenocarcinoma, immunocytochemical staining with anti‐AE1/AE3 antibody and endothelial markers, including anti‐CD31 antibody, anti‐ERG antibody, and anti‐FLI‐1 antibody, was performed on the cytology specimen (Table 1). The immunocytochemical results demonstrated the positivity of atypical cells for CK AE1/AE3, CD31, ERG, and FLI‐1, supporting the diagnosis of EAS (Figure 3). In this case, the diagnosis was facilitated through the combination of stamp cytology and ICC, complementing the gross findings from the surgically resected lung.

**TABLE 1:** Immunocytochemistry method.

	Anitigen retrieval (pH 9)	Dilution ratio/reaction time	Clone	Manufacturer
First antibody				
CD31	98°C, 5 min	1: 40/30 min	JC70A	Dako
ERG	98°C, 5 min	1: 50/30 min	EP111	Dako
Fli‐1	98°C, 5 min	1: 700/30 min	EPR4646	Abcam
CK AE1/AE3	None	Diluted antibody/30 min	AE1/AE3	Dako
Second antibody				
Histofine simple stain MAX‐PO (MULTI)		Diluted antibody/30 min		Nichrei

*Note:* Use 95% alcohol‐fixed cytological specimens. After antigen retrieval, keep at room temperature for 30 min.

**FIGURE 4:**
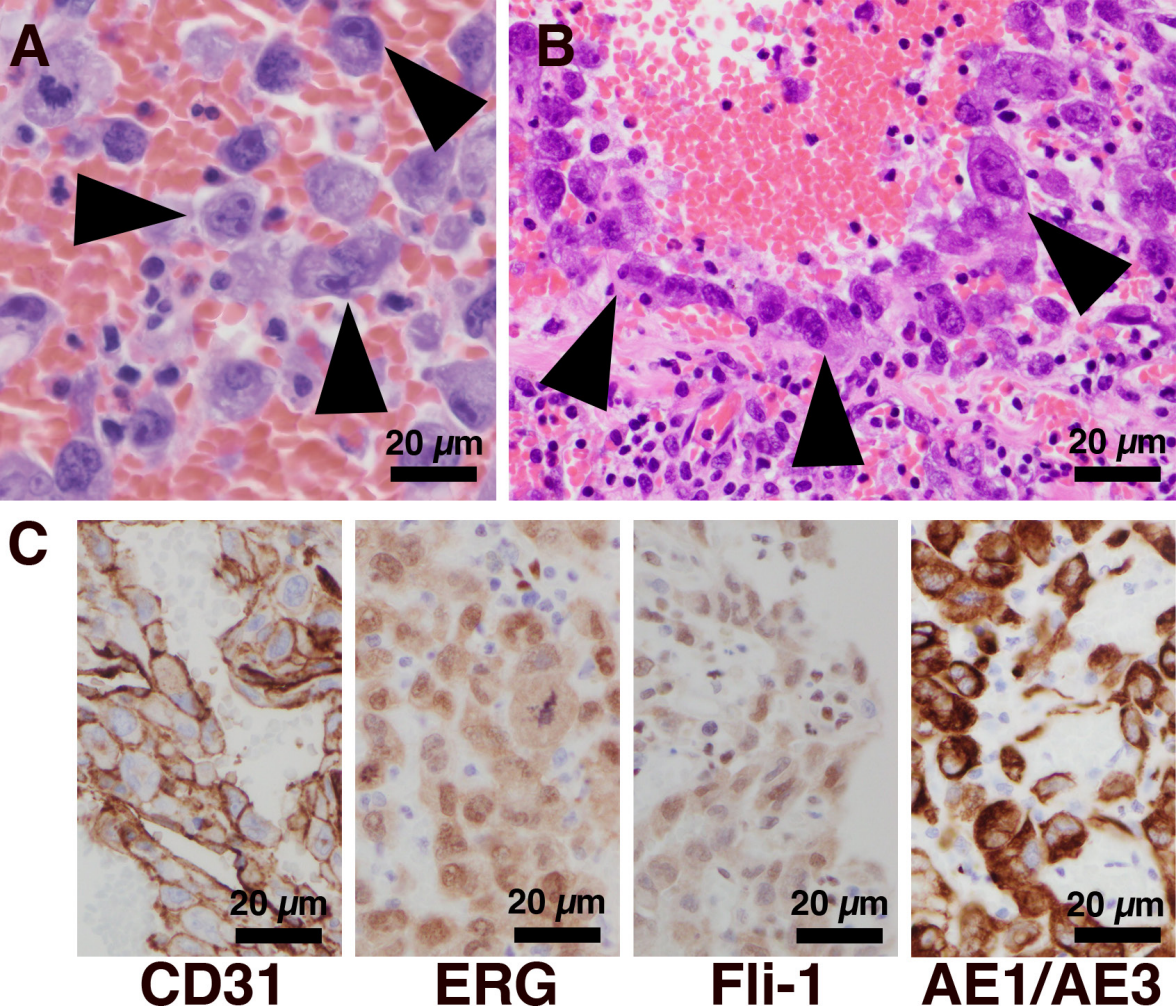
Histological findings in lung tumor. (A) Atypical cells with irregular nuclei and prominent nucleoli (arrowheads) in a hemorrhagic background. (B) Tumor cells forming a thin‐walled lumen with hobnail‐like atypical cells (arrowheads). (H&E) (C) Immunohistochemical findings. CD31 stain showing cell membrane positivity. ERG stain showing nuclear positivity. FLI‐1 stain showing nuclear positivity. Cytokeratin AE1/AE3 stain showing cytoplasm positivity.

The subsequent histological diagnosis confirmed angiosarcoma based on both histological and immunohistochemical evidence. The histological examination revealed the presence of atypical cells with irregular nuclei and prominent nucleoli in a hemorrhagic background (Figure 4A). The tumor cells exhibited the formation of thin‐walled lumens, within which hobnail‐like atypical cells were identified, and the lumens were filled with blood (Figure 4B). Immunostaining further supported the diagnosis by demonstrating the positivity of atypical cells for CD31, ERG, FLI‐1, and AE1/AE3 (Figure 4C). Consequently, these observations led to the establishment of a definitive diagnosis of EAS.

## 3 Discussion

We encountered a case where a vascular neoplasm was suspected based on the gross findings of the surgical specimen, and the diagnosis of EAS was established through a combination of touch imprint cytology and ICC. Previous studies have reported very few cases of EAS diagnosed solely on cytological specimens, including Geller et al. and Klijanienko et al. [9, 10]. This report highlights the potential for diagnosing EAS using cytological samples, which could be valuable in situations where obtaining tissue samples is not feasible.

While the cytological diagnosis of EAS is highly challenging, the ability to suggest its presence is crucial for achieving an accurate diagnosis. EAS is characterized by round to oval cells, polygonal shapes, epithelioid clusters, erythrophagocytosis, and backgrounds with hemorrhage and neutrophils [11]. Erythrophagocytosis is reported to be a less sensitive but highly specific finding [9]. Furthermore, Wakely, Frable, and Kneisl have reported the presence of cytoplasmic vacuoles [12, 13, 14]. In this case, all of these cellular findings were observed. Additionally, Liu and Layfield emphasize hemosiderin deposition in malignant cells as a diagnostic feature of angiosarcoma; however, it was not observed in this case [15]. Preoperative diagnosis of EAS based solely on cytological material is infrequent [10]. However, with careful consideration of clinical and imaging findings, we posit that a definitive diagnosis can be achieved through cytology and by correctly selecting antibodies for angiosarcoma diagnosis via immunostaining (Table 2) [9, 18].

**TABLE 2:** Literature review of cytological features and immunohistochemistry of epithelioid angiosarcoma.

Author	Year	Cytological features	Immunohistochemistry	
			Positive	Negative
Wakely [12]	2000	Cytoplasmic vacuoles	CD31	S‐100
		Multinucleates cells	CD34	HMB45
		Rhabdoid cells	Pancytokeratin	Actin
		Anisonucleosis	Vimentin	
			Factor 8	
Boucher [4]	2000	Oval to round to polygonal cells	CD31	
		Moderate pleomorphism; high N/C ratio	Cytokeratin	
		Some eosinophilic macronucleoli	Factor VIII	
		Abnormal mitoses	CD34	
		Cytoplasmic vacuoles	B72.3	
		Bloody background		
Gagner [16]	2005	Convoluted nuclei	AE1/AE3	CD31
			CD34	Factor VIII
			Vimentin	Calretinin
			CAM5.2	Desmin
			EMA	HMB‐45
				S‐100
Isa [14]	2009	Multinucleates cells	CD31	CD34
		Prominent nucleoli		Factor VIII
		Cytoplasmic vacuoles		CK5/6
		Inflammatory background		EMA
				Melan A
				Calretinin
Kuroda [8]	2009	Vasoformative structures	von Willebrand factor	Pancytokeratin
		Cytoplasmic vacuoles	CD31	S‐100
		Erythrophagocytosis	CD34	Desmin
			Fli‐1	SMA
Pohar‐Marinsek [17]	2009	Gland‐like structure	CD31	CD34
		Eccentric nuclei		
		Multinucleates cells		
		Cytoplasmic vacuoles		
		Anisonucleosis		
		Hemosiderin in the cytoplasm		
Sullivan [18]	2015	Convoluted nuclear membranes	ERG	
		Bar‐shaped nucleoli	CD31	
		Erythrophagocytosis	CD34	
			AE1/AE3	
Zhou [13]	2018	Prominent nucleoli	CD31	Melan A
		Cytoplasmic vacuoles	c‐MYC	HMB45
		Bar‐shaped nuclei	CAM5.2	CD34
			AE1/AE3	SMA
			Fli‐1	
			Vimentin	
Present case		Cytoplasmic vacuoles	AE1/AE3	EMA
		Prominent nucleoli	CD31	Desmin
		Erythrophagocytosis	ERG	CD34
		Bloody background	Fli‐1	S‐100

The key to diagnosing EAS through immunostaining lies in the careful selection of antibodies. Firstly, given the frequent expression of epithelial markers in EAS [6, 16, 18, 19, 20, 21, 22], they should be included in the immunostaining panel. In particular, CK AE1/AE3 is positive in many EAS cases [16]. Additionally, CD31, ERG, and Fli‐1 should incorporated as useful endothelial markers for EAS diagnosis [18]. Wu, Li, and Liu reported that CD31 and Fli‐1 exhibit greater sensitivity as markers for endothelial cells compared with CD34 and Factor VIII‐related antigen [22]. Sullivan et al. reported that ERG demonstrates comparable sensitivity to CD31 and is valuable in diagnosing angiosarcoma [18]. Establishing evidence of vascular differentiation in epithelioid tumor cells through positive staining for endothelial markers is particularly crucial for diagnosing EAS.

In this case, CK7 positivity was observed in immunohistochemistry (IHC) conducted during the biopsy. While Lin et al. reported CK7 positivity in thyroid EAS, to the best of my knowledge, this is the only report of CK7 positivity in EAS [23]. The significance of this CK7 positivity remains unclear, and accumulation of further cases is necessary to clarify this issue.

When performing the definitive diagnosis of EAS using cytological material, the reliability of ICC becomes crucial. Unlike IHC, ICC lacks established staining methods, which can lead to potential false negatives and false positives. To address this, IHC was also performed on tissue specimens from the same case. False negatives and false positives were eliminated by comparing staining characteristics between IHC and ICC, confirming specific staining by observing positivity with the same antibodies and similar cellular localization. Interestingly, ICC yielded clearer positive images compared with IHC (Table 3; Figures 3 and 4C).

**TABLE 3:** Characteristics of immunostaining.

Antibody	Staining intensity		Localization of positive staining
	Immunocytochemistry	Immunohistochemistry	
CD31	Strong	Strong	Cell membrane
ERG	Strong	Moderate	Nucleus
Fli‐1	Strong	Weak	Nucleus
CK AE1/AE3	Strong	Strong	Cytoplasm

The differential diagnoses for EAS include poorly differentiated carcinoma, melanoma, mesothelioma, epithelioid hemangioendothelioma (EHE), and other epithelioid sarcomas. Carcinoma typically shows negativity for endothelial markers, while malignant mesothelioma is negative for endothelial markers but positive for mesothelial markers such as calretinin and D2‐40. EHE typically exhibits positivity for CAMTA1 or TFE3 [24]. Melanoma is negative for endothelial markers but positive for melanocytic markers such as Melan‐A, S‐100, and HMB‐45. Our report underscores that with a comprehensive understanding of the cytological characteristics of EAS and the appropriate immunostaining panel, a reliable diagnosis of EAS can be achieved using cytological samples alone. Positive results for both endothelial and epithelial markers, even with a limited quantity of collected cells, significantly contribute to the diagnosis of EAS.

The most important aspect of this paper is that it details the method of immunocytochemical staining using cytology specimens for the diagnosis of angiosarcoma. Providing a detailed description of the immunocytochemical staining method is important to enable diagnosis based solely on cytology. While previous reports have described the results of immunocytochemical staining and methods of concurrent immunohistochemical staining, no papers have detailed the methods of immunocytochemical staining specifically for diagnosing angiosarcoma [8, 12, 17, 18, 22, 25]. By using this method, a definitive diagnosis can be made from cytology specimens even in cases where tissue sampling is difficult.

In conclusion, this report provides a diagnostic method for EAS using cytology. We prepared touch imprint cytology specimens from lung tumors excised during surgery and demonstrated the immunocytochemical staining method. When EAS is clinically and cytologically suspected, performing immunocytochemical staining using our described method enables a definitive diagnosis of EAS based solely on cytology.

## Author Contributions

**Tatsuya mori:** conceptualization (equal), investigation (equal), writing – original draft (lead). **Keishi Mizuguchi:** conceptualization (equal), methodology (lead), writing – review and editing (lead). **Chie Shimaguchi:** investigation (equal). **Kaori Sakano:** investigation (equal). **Tsubasa Shimoda:** investigation (equal). **Urara Okawa:** investigation (equal). **Miyu Okuda:** investigation (equal). **Mayo Usui:** investigation (equal). **Hiroko Ikeda:** supervision (lead).

## Ethics Statement

The study adhered to the tenets of the Declaration of Helsinki. The retrospective case report described in this manuscript includes no patient‐identifying information. Ethical approval was not required.

## Consent

We obtained a written statement of informed consent from the patient for the publication of case details and the use of images.

## Conflicts of Interest

The authors declare no conflicts of interest.

## Data Availability

The data that support the findings of this study are available from the corresponding author upon reasonable request.
